# Background selection for camouflage shifts in accordance with color change in an intertidal prawn

**DOI:** 10.1093/beheco/arae060

**Published:** 2024-07-27

**Authors:** Samuel D Green, Alastair Wilson, Martin Stevens

**Affiliations:** Centre for Ecology and Conservation, University of Exeter, Penryn Campus, Cornwall TR10 9FE, United Kingdom; Centre for Ecology and Conservation, University of Exeter, Penryn Campus, Cornwall TR10 9FE, United Kingdom; Centre for Ecology and Conservation, University of Exeter, Penryn Campus, Cornwall TR10 9FE, United Kingdom

**Keywords:** behavioral background matching, camouflage, Caridea, coloration, plasticity

## Abstract

To maximize camouflage across visually heterogeneous habitats, animals have evolved a variety of strategies, including polyphenism, color change, and behavioral background matching. Despite the expected importance of behavioral processes for mediating camouflage, such as selection for matching substrates, behavior has received less attention than color traits themselves, and interactions between color change and behavior are largely unexplored. Here, we investigated behavioral background matching in green and red chameleon prawns (*Hippolyte varians*) over the course of a color change experiment. Prawns were housed on mismatching green and red seaweeds for 30 days and periodically given a choice test between the same seaweeds in y-choice trials over the experiment. We found that, as prawns change color and improve camouflage (to the perspective of a fish predator), there is a reinforcing shift in behavior. That is, as prawns shift from red to green color, or vice versa, their seaweed color preference follows this. We provide key empirical evidence that plasticity of appearance (color) is accompanied by a plastic shift in behavior (color preference) that reinforces camouflage in a color changing species on its natural substrate. Overall, our research highlights how short-term plasticity of behavior and longer-term color change act in tandem to maintain crypsis over time.

## Teaser

Many animals change color for camouflage. Similarly, individuals can behaviorally choose substrates that match their color. However, as an animal changes to match a new background its behavioral preference should change too. We demonstrate that red and green variants of the chameleon prawn undergo simultaneous changes in color and background preference for camouflage against seaweeds. Such synergy between physiological and behavioral changes is likely to be critical to successful camouflage in animals inhabiting variable environments.

## Introduction

For visual camouflage to be effective there must be a close relationship between animal phenotype and aspects of the visual environment, i.e. phenotype–environment matching (Stevens and Merilaita 2009; Merilaita et al. 2017; Cuthill 2019). A challenge for mobile species relying on camouflage is that microhabitats (and the colors, patterns, and textures within them) vary over time and space, therefore animals require strategies to maintain camouflage as they move through their environment (Caro et al. 2016). These strategies may be generated by coloration itself, such as generalist (or “compromise”) camouflage forms and individual variation (e.g. polymorphism and polyphenism) (Nachman et al. 2003; Ahnesjö and Forsman 2006; Hughes et al. 2019; Briolat et al. 2021), or more flexible strategies, such as color change (Hanlon 2007; Stuart-Fox and Moussalli 2009; Gilby et al. 2015; Duarte et al. 2017; Eacock et al. 2017). In addition, animals may improve camouflage through behaviors, e.g. choosing camouflage-appropriate backgrounds that match individual appearance (Kettlewell 1955; Hacker and Madin 1991; Kang et al. 2012; Lovell et al. 2013; Marshall et al. 2016; Polo-Cavia and Gomez-Mestre 2017; Stevens and Ruxton 2019).

Behavioral background matching (hereafter “behavior”) involves animals having background preferences that complement their coloration within the visual environment, thereby improving camouflage (Kang et al. 2012; Lovell et al. 2013; Marshall et al. 2016; Stevens and Ruxton 2019; Camacho et al. 2020). Despite the intuitiveness of this idea, and a relatively long history of experiments in certain taxa such as moths (e.g. Kettlewell 1955), there has generally been a lack of rigorous experimentation of how and when behavioral choices facilitate crypsis on natural substrates (Stevens and Ruxton 2019). To gain a more comprehensive understanding of how the fitness benefits of camouflage are maintained, it is important to test to what extent organisms combine choice of visually appropriate backgrounds with other strategies, because it is likely that behavioral and morphological phenotypes have coevolved in species relying on camouflage (Kang et al. 2012; Uy et al. 2017). Correspondingly, a significant gap in research involves testing if, and when, behavioral preferences are flexible, and how this links with other aspects of camouflage such as color change.

Various studies across taxa, incorporating ecologically relevant backgrounds have demonstrated that behaviors can complement animal appearance to improve camouflage. For example, the chosen resting spots of Aegean wall lizards (*Podarcis erhardii*) (Marshall et al. 2016) and nesting substrate preferences of Japanese quail (*Coturnix japonica*) (Lovell et al. 2013) demonstrate individual-specific camouflage benefits. In ground-nesting birds, individuals can choose appropriate backgrounds in relation to either adult plumage or egg pigmentation, depending on brood-defense strategy (Stevens et al. 2017). For polymorphic/polyphenic species it is expected that intraspecific variation in coloration may be mirrored by variation in behavior. Broadly speaking this has been found to be the case, although there seems to often be inconsistencies in behavioral preferences between morphs and/or individuals. For example, the Pacific tree frog (*Hyla regilla*) is found in fixed green and brown forms, in addition to a morph capable of color change between brown and green. Of the fixed color morphs, only green individuals show a background preference, whereas fixed brown and color changing frogs showed no preference (Wente and Phillips 2003). Most studies to date have tested background choices in species where individuals are fixed in appearance. For species capable of color change it is important to consider how behavioral preferences may be closely linked to phenotypic appearance. The duration of color change is highly variable in nature, and it would likely be maladaptive for the behavioral preferences of species to not correspondingly shift with color phenotype.

Color change for camouflage allows species to directly alter appearance in relation to temporal and spatial variation in the visual environment (Stevens 2016a; Duarte et al. 2017). It is particularly common among poikilothermic vertebrates and invertebrates, where networks of specialized chromatophore cells affect coloration over a range of timescales through varying pigment within the cells, or the distribution and synthesis of the cells themselves (Bagnara and Hadley 1973; Umbers et al. 2014; Stevens 2016b). Intuitively, species capable of rapid color change might be under less intense selective pressure to rely on behaviors for camouflage, as they could instead simply alter appearance in relation to background variation. For example, cuttlefish (*Sepia officinalis*) generally do not exhibit substrate preferences but rely on visual environmental cues to adopt camouflage patterns (Allen et al. 2010). However, individuals from species that change color over a longer duration may still strongly depend on an ability to select visually appropriate backgrounds to maintain camouflage, potentially also reducing the level of mismatch during the process of color change (Caro et al. 2016; Stevens and Ruxton 2019).

The relationship between behavior and color change likely depends on several factors. Rock gobies (*Gobius paganellus*), e.g. rapidly alter coloration to improve camouflage, but individuals still select darker backgrounds that improve matching, indicating that a combination of behavior and color change may reduce limitations of each strategy (Smithers et al. 2018). The expression of behaviors that improve camouflage may also vary between color forms. Filefish (*Monacanthus chinensis*), e.g. are found in brown and green forms, but only brown fish display color change and show substrate preference (Gilby et al. 2015). The authors suggested that green forms may need to forage across more habitats, therefore the green form may constitute some form of generalist camouflage. Overall, color change and oriented-choice behavioral traits will be expected to be under strong selection pressure to combine to maximize the level of camouflage a species experiences (Magellan and Swartz 2013; Smithers et al. 2018; Stevens and Ruxton 2019). Given that rapid color change (in seconds) is much less common than slower changes occurring over minutes, hours, and days, many color changing animals may, therefore, rely on behavioral choice to maintain camouflage in the short to medium term. However, this also poses a potential problem, because as an animal changes color to match a new background we would expect a corresponding switch in behavioral preference for this new background too. Optimally, this switch should occur at a point in time that the animal starts to better match the new background, or to reinforce a preference for a background that the animal is beginning to resemble.

**Fig. 1.**
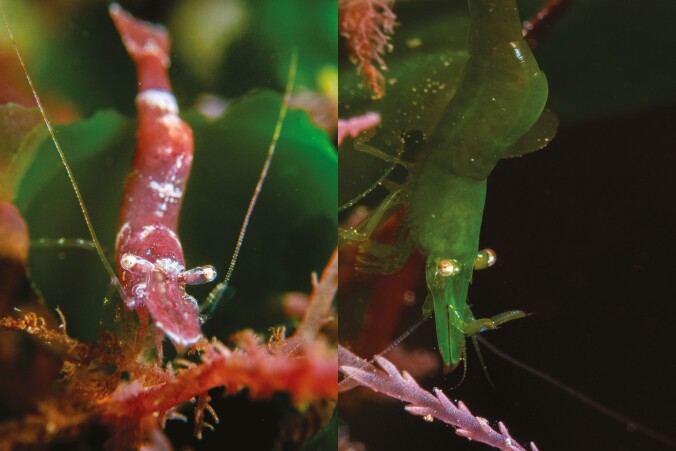
Photographs of the red and green variants of chameleon prawns. The green seaweed in the background is sea lettuce, whereas the pink foreground weed in both photos is an invasive harpoon weed (which red prawns also like to rest on).

Here, we study if and how preferences for matching backgrounds switch alongside color change in the chameleon prawn (*Hippolyte varians*). This species is highly variable in appearance (Fig. 1) and color types are well camouflaged against specific seaweed backgrounds. In the short term, color variants choose camouflage-appropriate substrates, but they also change color over a period of 2–3 weeks to reduce mismatch against contrasting seaweeds (Gamble and Keeble 1900; Chassard 1956; Green et al. 2019). Substrates within the intertidal zone where the prawns live are visually highly variable in space and time, and so both behavior and color change are key to maintaining concealment. We tested how the background preferences of green and red prawns changed over the course of a 30-day color change experiment. We expected that as prawns change color to match seaweed substrates their behavioral preferences would change in tandem. Our results show that, as both color types change color and improve camouflage, their behavioral preferences shift in the same direction as their coloration.

## Materials and methods

### Collection and husbandry

Green and red color forms of the chameleon prawn (*Hippolyte varians),* and correspondingly colored seaweeds—sea lettuce (*Ulva lactuca*) and dulse (*Palmaria palmata*)—were sampled from rockpools at low tide on Gyllyngvase beach, Falmouth, Cornwall, United Kingdom (50°08ʹ33″N, 05°04ʹ08″W) in the summer of 2019 (Fig. 1). For details on laboratory husbandry see Supplementary Materials. This work was approved by the University of Exeter Bioscience Ethics Committee (2017/1568).

### Photography and visual modeling

Green (*n* = 58) and red (*n* = 57) chameleon prawns were held in the lab for a 30-day period and the general approach and setup followed (Green et al. 2019). Having acclimatized to tank conditions on their associated seaweed, prawns were switched to mismatching substrates (green prawns to dulse, red prawns to sea lettuce) and photographed (day 0). Prawns were then photographed on days 15 and 30 to track color change over the experimental period. Seaweed samples that the individual prawns were housed on were photographed under the same conditions.

A Nikon D90 SLR camera with a 105 mm Nikkor lens fitted with a Baader UV–IR blocking filter (Baader Planetarium, Mammendorf, Germany), permitting only visible spectrum light (420–680 nm), was used to record coloration. Illumination was provided by an Arc Lamp (70W, 6500K Iwasaki Color Arc Lamp, Tokyo, Japan) that had been modified by removing its UV filter. Prawns and seaweeds were photographed submerged in a custom acrylic chamber with a PTFE diffuser to ensure even lightning conditions and reduce specular reflections. Images were taken in RAW format with manual white balancing and fixed aperture settings to reduce over exposure of pixels (Stevens et al. 2007) and included a dual reflectance standard (Labsphere, North Sutton, USA: 7% and 93%). Using ImageJ (Schneider et al. 2012) and a series of customized plugins, photographs were linearized to correct for nonlinear camera responses to lighting intensity, and any variation in lighting intensity between images was controlled for by the reflectance standard (Stevens et al. 2007; Troscianko and Stevens 2015), resulting in a 32-bit multispectral image. Spectral sensitivity of the camera in combination with the lens and visible light filter had previously been characterized (Troscianko and Stevens 2015).

We chose to use the 2 spotted goby (*Gobiusculus flavescens*) in the predator visual model, a trichromatic species with peaks at 456 nm (SWS) for their single cone in addition to 531 nm (MWS) and 553 nm (longwave sensitivity—LWS) for double cones (Utne-Palm and Bowmaker 2006). A D65 standard irradiance spectrum was used as a measure of incident illumination for the models (Wyszecki and Stiles 2000). Cone catch values for goby vision were produced by converting images from camera color space to fish vision with a polynomial mapping function built into the image analysis toolbox (Westland and Ripamonti 2004; Stevens et al. 2007; Troscianko and Stevens 2015). The ultimate result of the visual modeling was multispectral images where predicted cone catch data for each cone type could be extracted from the image in defined regions of interest (ROIs). Prawn ROIs consisted of the carapace from just behind the eyes to the start of the tail fans although seaweed ROIs consisted of the entire sample.

### Color analysis

Hue and “just noticeable differences” (JNDs) were used to quantify color change and camouflage match against seaweeds, respectively. Hue, a ratio of visual system specific cone catch values, had been previously defined for goby vision to measure variation in prawn and seaweed coloration (Green et al. 2019). Therefore, hue is defined as [SWS/MWS + LWS]: this ratio measures shortwave reflectance against that of the other color channels. JNDs are values arising from a widely used receptor noise model for discriminating between color samples, integrating Weber fractions based on the cone ratios of visual models and a noise-to-signal ratio of 0.05 for the most abundant MWS cone type (Vorobyev and Osorio 1998; Utne-Palm and Bowmaker 2006; Olsson et al. 2018). The resulting values are a numerical scale where, as JNDs increase, color samples are predicted to be less similar. Using this method, color measurements of prawns and seaweeds were compared over the 30-day experiment.

### Behavioral trials

Alongside the color change experiment, the behavioral preferences of prawns for seaweed types were tested using custom made choice chambers (see Supplementary Materials). Samples of dulse and sea lettuce were placed in separate arms and alternated (left/right) between trials. In the trials, prawns were acclimatized to the chambers for 1 min with line of sight to seaweeds obscured. Trials lasted for 10 min with first choice being defined as the first seaweed that prawns spent > 10 s associating with following (Green et al. 2019). We consider this “first choice” metric appropriate as for species relying on camouflage the initial decision is likely to be key to survival (e.g. if a prawn was dislodged by wave action) and prawns tend to sit closely on seaweed once they have chosen. Prawn decision within the allotted time was observed in 211 of 241 trials (excluding a small subset of prawns where data could not be collected—see Supplementary Materials) and the likelihood of making a choice did not differ between the 2-color types (*X*^2^ = 0.25, df = 1, *P* = 0.62). Final sample sizes where choice occurred were (green/red prawns) days 0 (39/41), 15 (48/48), and 30 (15/20).

### Statistical analysis

All statistical analyses conducted here used R v. 3.5.1 (R Development Core Team 2016) in R Studio v. 1.3.1073 (RStudio Team 2016). Color change was analyzed separately for each prawn color form. Hue and JND were independently analyzed using linear mixed effects models including day as a numerical fixed effect and a random intercept of prawn ID to account for repeated measurements of the same individual across days. Linear models were fitted using the “lmer()” function in the package lme4 (Bates et al. 2015). Model residuals were checked visually for normality and homogeneity of variance. There were no deviations deemed major enough departures from the normal assumptions to necessitate data transformation. Inference on fixed effects was performed using the lmerTest function (ANOVA with Satterthwaite’s method) generating *P* values (Kuznetsova et al. 2017). Behavioral substrate preferences for both color types during color change were analyzed using a generalized linear mixed effect model with a binomial error structure using the package “afex” which applies a logit link function (Singmann et al. 2021), with significant outcomes of this reported as Chi-square tests. The “mixed()” function allows for binomial models to be fitted using “lmer()” within “lme4,” with likelihood ratio tests used to generate chi-squared statistics for inference on explanatory variables. The dependent variable choice was the type of seaweed chosen by prawns coded as sea lettuce (0) or dulse (1). Day (numerical—0, 15, and 30) and prawn color (factor—green or red) were included as fixed explanatory effects. In addition, to test for differences in the effect of body color on substrate preference over time, the interaction between color and day (color:day) was included in the model. NAs (where there was no choice) were manually removed from the behavior dataset using the “filter” command in the dplyr package (Wickham et al. 2021). We predicted that prawns of both color types would change color to improve camouflage and that as these changes occurred behavioral substrate preferences would change in tandem with coloration. Specifically, we expected green prawns to increase in hue (become redder) and red prawns to reduce in hue (become more green). We expected that green prawns would change to prefer red substrates, and red prawns to prefer green substrates, as they changed color type.

## Results

**Fig. 2.**
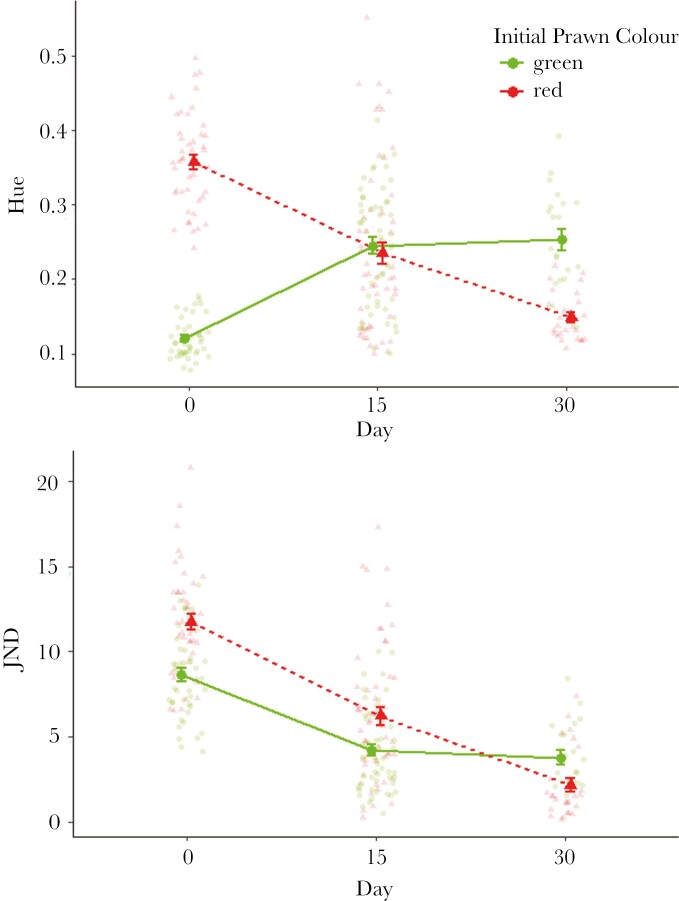
Color change improves chameleon prawn camouflage in response to mismatched seaweed. Top) Changes in hue (mean ± 95% CI from the statistical model) of green and red prawns when placed against seaweed of opposing coloration over 30 days to modeled goby vision. A clear crossover can be observed between green and red prawns over time due to the increase and decrease respectively in the proportion of SW reflectance. Bottom) Changes in JNDs (mean ± SE) over time for green and red prawns to the modeled perspective of goby vision, showing that both green and red prawns change in response to new substrates, which improves the level of camouflage (lower JNDs).

First, to quantify color change the hue of green and red prawns was recorded over 30 days. Hue values of green prawns kept on dulse significantly increased over time to goby vision (LMM: *F*_*1,* 74_ = 114.01, *P* < 0.01—Fig. 2a). Conversely, hue of red prawns maintained on sea lettuce significantly decreased over time to goby vision (LMM: *F*_*1, 64*_ = 145.88, *P* < 0.01—Fig. 2a). Changes in hue are primarily a result of changes in the proportion of shortwave (SW) reflectance over time to predator vision. Ultimately, green prawns kept on red dulse became redder, whereas red prawns kept on green sea lettuce became greener. Again, corresponding with previous findings (Green et al. 2019), changes in hue were more apparent in the early stage of the experiment (days 0–15: Tukey *t*-tests; *P* < 0.001) compared with the later period, particularly for the green prawns (days 15–30: Tukey *t*-tests; Green prawns *P* > 0.4, red prawns *P* < 0.01).

To test if change in color translated into an improvement of camouflage, color JNDs were calculated between prawn and host seaweed coloration. JNDs between prawn color types and mismatching seaweeds significantly reduced over time based to goby vision (LMM—green prawns: *F*_1, 79_ = 86.43, *P* < 0.01, day 0 = 8.64 ± 0.38 → day 30 = 3.77 ± 0.40; red prawns: *F*_1, 71_ = 220.84, *P* < 0.01, day 0 = 11.70 ± 0.47 → day 30 = 2.15 ± 0.42, Fig. 2b). Overall, these results demonstrate that coloration between prawns and the (initially) mismatching seaweeds become more visually similar over the experimental period, improving camouflage to predator vision. Post hoc testing showed that changes in JNDs were only significant in the first half of the experiment for green prawns (Tukey *t*-tests: days 0–15; *P* < 0.0001, days 15–30; *P* = 0.57) whereas red prawns continued to show a significant reduction in JNDs throughout the experiment (Tukey *t*-tests: days 0–15; *P* < 0.0001, days 15–30; *P* < 0.0001).

**Fig. 3.**
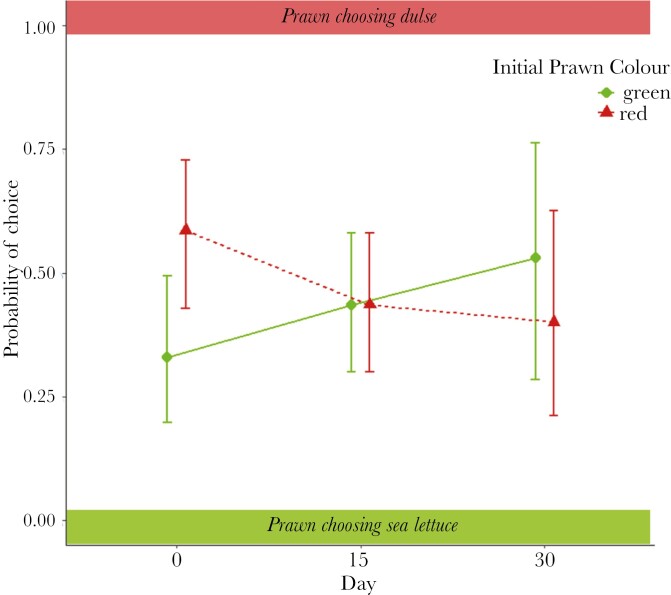
Behavioral choice shifts in tandem with color change. Changes in the probabilities (mean ± 95% CI from the statistical model) of green and red prawns choosing between dulse and sea lettuce over the 30-day color change experiment. Over the course of the experiment the probability of “green” prawns choosing dulse and “red” prawns choosing sea lettuce increased, corresponding to changes in coloration improving camouflage to predator vision.

To assess if the substrate preferences of prawns change in tandem with a change in coloration, behavioral choice trials between sea lettuce and dulse were conducted over the color change period. At the beginning of the experiment there was a significant difference in the substrate preferences of green and red prawns (*X*^2^ = 5.15, df = 1, *P* = 0.02, Fig. 3), with a higher probability of green prawns preferring sea lettuce and red prawns preferring dulse. There was also a significant interaction between day and color type (*X*^2^ = 4.31, df = 1, *P* = 0.04), such that the probability of substrate preference changed over time depending on the initial coloration of prawn types. This change resulted in initially green prawns displaying an increasing probability of choosing the red dulse (probability ± SE of choosing dulse; days 0–0.33 ± 0.08, days 15–0.44 ± 0.07, days 30–0.53 ± 0.13). Conversely, initially red prawns show an increasing probability of choosing the green sea lettuce substrate (probability ± SE of choosing sea lettuce; days 0–0.41 ± 0.08, days 15–0.56 ± 0.07, days 30–0.60 ± 0.11) as color change occurred (Fig. 3). There was no significant interaction affecting the probability of choice between hue and day (*X*^2^ = 1.11, df = 1, *P* = 0.29) or JND and day (*X*^2^ = 1.23, df = 1, *P* = 0.27). Overall, these results show that, for both color types, behavioral preferences switched alongside a change in color for camouflage, but that the probability of choice was not affected by coloration or the level of camouflage of individual prawns.

## Discussion

Our results show how the close association between behavior and color change maximizes concealment for chameleon prawn color forms against natural backgrounds. Using green and red color forms and a model of predatory trichromatic fish vision we quantified changes in coloration and camouflage against green sea lettuce and red dulse. Our findings support those of earlier work (Green et al. 2019), whereby prawns can change color against contrasting seaweeds to improve camouflage over a 30-day period. However, in this study we also tested changes behavioral preferences between color forms and seaweeds during color change, demonstrating that seaweed preference shifts in the direction of coloration during color change. These findings clearly demonstrate how plastic camouflage traits concerning coloration and behavior work collaboratively to maximize camouflage in space and time.

The color variation in polymorphic or polyphenic species provides ideal systems with which to test hypotheses regarding adaptive interactions between behavior and coloration for camouflage. Although morph-specific behaviors have been observed across a range of taxa, choice is often inconsistent between morphs and individuals, and understanding why these differences occur remains a largely underexplored topic (Stevens and Ruxton 2019). An example being how intraspecific variation in coloration and behavior may allow individuals to utilize distinct aspects of visual camouflage to adopt alternative life histories (Kusche et al. 2015). This is apparent in “carnival” prawns, where substrate preferences and morph-specific differences in morphology and mobility suggest contrasting benthic/pelagic lifestyles (Duarte et al. 2016). Further, behaviors improving camouflage can drive phenotype–environment associations in wild populations. For example, azure sand grasshoppers have been found to be naturally distributed across camouflage-appropriate areas of pavement and, furthermore, when coloration was artificially manipulated this pattern was repeated after recapture (Camacho et al. 2020). If species can adjust their behavior when appearance is artificially manipulated it is of great interest to better understand how behaviors correlate with plastic color traits, such as color change (it is worth noting that adult grasshoppers were able to change color, but not in timescales relevant to their work).

Although research has demonstrated that species can use combinations of color change and behavior to maximize concealment over variable timescales (e.g. Smithers et al. 2018; Green et al. 2019), there has been little inquiry into how animals adapt behaviors during changes in coloration. This is important, because if behavioral choice is out of sync with color change then animals may continue to choose backgrounds that they no longer match, or inappropriate choice may impede effective change if color change depends on consistent visual feedback from background sensory features. Although Trinidadian guppies (*Poecilia reticulata*) have been shown to mediate behavioral preferences between artificial black and white habitat zones when induced to change color in correspondingly colored tanks (Rodgers et al. 2013). The data presented here provides strong empirical evidence of behaviors changing in tandem with a longer-duration change in coloration, where relative camouflage levels are quantified against the natural substrates on which the animal lives. The changes in coloration displayed here by green and red prawns improve camouflage to predator vision against the mismatching substrates on which they are housed. Critically, over the course of the color change experiment the prawn’s behavioral substrate preferences shift in same the direction as color change. This would enable prawns to actively maintain the fitness benefits of cryptic coloration regardless of the state of phenotypic expression.

Phenotypic integration between behavioral and color traits is likely dependent on multiple factors. As previously highlighted, species that rapidly adjust coloration are predicted to be less dependent on behaviors to maintain crypsis. *Galaxias* ‘nebula’ individuals, for example, can alter pigmentation over a few minutes and show no behavioral preferences but vary the expression of dark pigment, negating the need for background selection (Magellan and Swartz 2013). Arguably, any change taking longer than seconds/minutes may benefit from supporting behavioral adaptations as predation events only need a short period of time in which to occur, and any degree of mismatch would make an animal vulnerable. Ryer et al (2008) demonstrated that north Pacific flatfish species that can change color over a period of days choose camouflage-appropriate substrates. However, when isolated on mismatching backgrounds, rather than relying on cryptic self-burying behaviors fish increased activity levels (Ryer et al. 2008). This would likely result in an increased predation risk but highlights the importance of actively searching for more appropriate substrates when color change is not temporally possible. In addition to the duration of change impacting camouflage efficacy, species may also have phenotypic limitations to color change. The peacock flounder (*Bothus lunatus*) can alter coloration in a matter of seconds but has a limited pattern repertoire, therefore strong behavioral preferences linked to the use of a range of microhabitats throughout coral reef systems are favored to maximize camouflage (Tyrie et al. 2015).

In the chameleon prawn, it is not currently known what cues individuals use to guide both color change and substrate choice. Although visual cues such as color or brightness may seem most intuitive, in practice prawns may also rely on other indirect means of choosing suitable backgrounds. Perhaps the most likely of these would be the use of chemical cues, in part because prawn diet is likely to include the seaweed itself, and because we have some provisional (unpublished) data suggesting that interference from artificial chemicals may disrupt correct substrate choice. In moths, substrate selection and alignment for visual camouflage can involve nonvisual (e.g. textural) information (Sargent and Keiper 1969; Kang et al. 2014). It is also possible that visual cues beyond color or brightness may be important; for example, some species of shrimp select substrates based on shape (Barry 1974). Future work would benefit from a better understanding of the mechanisms through which choice and color change are guided.

The challenges of remaining camouflaged in variable natural habitats are multifaceted. Although color-based strategies are thought to have evolved to help mitigate this challenge, coloration and behavior are not mutually exclusive traits and behaviors help to ecologically integrate color traits into the natural world (Stevens and Ruxton 2019). Our data provides strong empirical evidence of plasticity in background choice behavior for a species capable of color change to maintain camouflage, against natural substrates. Significantly, for a slow color changing species like the chameleon prawn, we have demonstrated that as the camouflage benefit of coloration switches between seaweeds so do behavioral preferences. This highlights the importance of behaviors for maintaining camouflage over timescales when color change is too slow to be adaptive. There are a growing number of studies testing how combinations of color and behavioral traits influence crypsis. Here, we provide data demonstrating that species may rely on multiple flexible camouflage strategies to maintain crypsis in heterogeneous natural environments and that these flexible strategies have likely evolved in close association to maximize camouflage in time and space.

## Supplementary Material

arae060_suppl_Supplementary_Materials

arae060_suppl_Supplementary_Data

## Data Availability

Analyses reported in this article can be reproduced using the data provided by Green et al (2024)
