# Supplementary material for: Background selection for camouflage shifts in accordance with color change in an intertidal prawn
Source: Behav Ecol. 2024 Jul 27;35(5):arae060. doi: 10.1093/beheco/arae060 (PMC11453103; doi:10.1093/beheco/arae060)
Supplement: arae060_suppl_Supplementary_Materials [file arae060_suppl_supplementary_materials.docx]

Methods:

Husbandry

Prawns were taken to the lab and transferred to controlled tanks. The salinity within the tanks was kept ~30 ppm using Aquarium Systems Instant Ocean Sea salt mixture (Swell Ltd, Cheshire, UK) and maintained at a constant temperature (matching local sea temperature ~14 °C) with a Swell D&D DC300 Refrigerated Cooler (Swell Ltd, Cheshire, UK). The water was oxygenated and filtered using an Eheim Classic 350 2215 External Filter (EHEIM GmbH & Co. KG, Germany). Natural lighting was simulated using two GroBeam 600 Ultima ND Natural Daylight and one AquaBeam 600 Ultima NUV (Tropical Marine Centre, Hertfordshire, UK) on a 12-hour light cycle from 07:30 to 19:30. Prawns were individually kept in 250ml clear labelled plastic tubs (Plastic Test Tubes Ltd, UK) within the larger tanks (Fig. 1). Black pot lids were drilled to remove a six-centimetre diameter circle leaving just the threaded outer rim, which was used to fasten ultra-fine insect netting over the top of the container. One-millimetre holes were drilled around the bottom edge of the container. These modifications allowed ‘natural’ tank lighting and increased water flow within the pots. The bottom centimetre of each pot was filled with natural coloured ‘Nordic’ 2-4mm aquarium gravel (Swell Ltd, Cheshire, UK). Prawns were initially kept on seaweeds matching their own coloration (green – sea lettuce, red – dulse) to acclimatise to the tanks. The work was approved by the University of Exeter Bioscience ethics committee (Code 2017/1568).

**
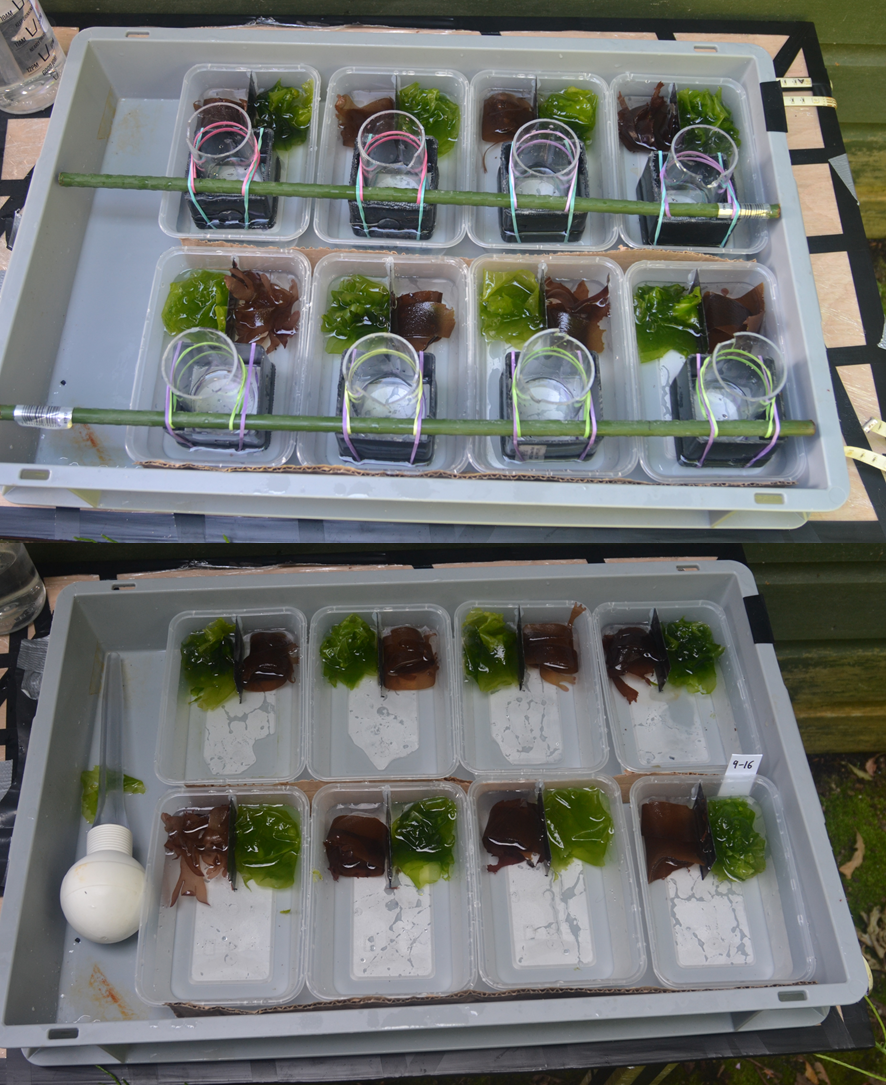
**Choice chambers

Constructed from 17.5x12x5cm plastic containers that were customised by adding a 7cm opaque divider to create the ‘arms’.

Missing Data

A memory card malfunction resulted in the photographs and videos for day 0 of half of the 2^nd^ batch (16 green and 15 red prawns) being unable to be processed and did not contribute data. Seaweeds were changed at a regular interval to ensure the substrates remained fresh throughout the experiment. However, due to unforeseen travel arrangements seaweeds were not changed between the day 15 and 30 photographs in the 2^nd^ batch and seaweed quality had deteriorated partly due to accompanying warm weather conditions. For this reason, the day 30 data of these prawns (28 green and 27 red, eight and two of which had died respectively) were excluded from the colour change and behaviour datasets as any changes in colour and/or behaviour may have been due to the above husbandry limitation leading to inferior substrate quality. Overall, five prawns (four green, one red) died between the day 0 and 15 photographs and a subsequently between days 15 and 30 a further five green and four red prawns died. This resulted in 91% of green and 98% of red prawns surviving between day 0 and 15, where colour change is again observed to be most abrupt (Fig. 6). Survival then dropped to 70% for green and 83% red prawns at the end of the experiment (excluding 2^nd^ batch prawns here which had 68% and 93% survival respectively). As can be seen, this species can be very sensitive to (presumed) changes to water chemistry when in captivity and, while not ideal, these levels of mortality are not unexpected in this system. Missing values (i.e. data for prawns that died or were excluded) were controlled using the option na.omit of the lmer function (see below), which allowed the inclusion of data from missing prawns in the colour change model but keeping only those from the time periods prawns contributed to.
